# The impact of perioperative complications on favorable outcomes after artificial urinary sphincter implantation for post-prostatectomy incontinence

**DOI:** 10.1590/S1677-5538.IBJU.2019.0526

**Published:** 2020-03-25

**Authors:** Alexander Kretschmer, Tanja Hüsch, Ralf Anding, Tobias Pottek, Achim Rose, Werner Struss, Fabian Queissert, Carsten M. Naumann, Joanne N. Nyarangi-Dix, Bernhard Brehmer, Axel Haferkamp, Ricarda M. Bauer

**Affiliations:** 1 Department of Urology Ludwig-Maximilians-University Campus Großhadern Munich Germany Department of Urology, Ludwig-Maximilians-University, Campus Großhadern, Munich, Germany;; 2 University Medical Center Johannes-Gutenberg University Mainz Germany University Medical Center of Johannes-Gutenberg University, Mainz, Germany;; 3 Department of Urology and Pediatric Urology University Hospital Bonn Bonn Germany Department of Urology and Pediatric Urology, University Hospital Bonn, Bonn, Germany;; 4 Department of Urology Vivantes Hospital Berlin Berlin Germany Department of Urology, Vivantes Hospital Berlin, Berlin, Germany;; 5 Department of Urology Helios Hospital Duisburg Duisburg Germany Department of Urology, Helios Hospital Duisburg Duisburg, Germany;; 6 Department of Pediatric Urology Helios Hospital Duisburg Duisburg Germany Department of Pediatric Urology, Helios Hospital Duisburg Duisburg, Germany;; 7 Department of Surgery, Urology University Hospital Southampton NHS Foundation Trust Hampshire United Kingdom Department of Surgery, Urology University Hospital Southampton NHS Foundation Trust, Hampshire, United Kingdom;; 8 Department of Urology University Hospital Southampton NHS Foundation Trust Hampshire United Kingdom Department of Urology, University Hospital Southampton NHS Foundation Trust, Hampshire, United Kingdom;; 9 Department of Urology University Hospital Muenster Muenster Deutschland Department of Urology, University Hospital Muenster, Muenster, Deutschland;; 10 Klinik für Urologie und Kinderurologie Marienhausklinikum Bendorf-Neuwied-Waldbreitbach Germany Klinik für Urologie und Kinderurologie. Marienhausklinikum Bendorf-Neuwied-Waldbreitbach, Germany;; 11 Department of Urology University Hospital Heidelberg Heidelberg Germany Department of Urology, University Hospital Heidelberg, Heidelberg, Germany;; 12 Diakonie Hospital Schwäbisch Hall Department of Urology Diakonie Hospital Schwäbisch Hall Schwäbisch Hall Germany Department of Urology, Diakonie Hospital Schwäbisch Hall, Schwäbisch Hall, Germany

**Keywords:** Urinary Incontinence, Stress, Urinary Sphincter, Artificial, Quality of Life

## Abstract

**Objective:**

To investigate the effect of perioperative complications involving artificial urinary sphincter (AUS) implantation on rates of explantation and continence as well as health-related quality of life (HRQOL).

**Materials and methods:**

Inclusion criteria encompassed non-neurogenic, moderate-to-severe stress urinary incontinence (SUI) post radical prostatectomy and primary implantation of an AUS performed by a high-volume surgeon (>100 previous implantations). Reporting complications followed the validated Clavien-Dindo scale and Martin criteria. HRQOL was assessed by the validated IQOL score, continence by the validated ICIQ-SF score. Statistical analysis included Chi (2) test, Mann-Whitney-U test, and multivariate regression models (p <0.05).

**Results:**

105 patients from 5 centers met the inclusion criteria. After a median follow-up of 38 months, explantation rates were 27.6% with a continence rate of 48.4%. In the age-adjusted multivariate analysis, perioperative urinary tract infection was confirmed as an independent predictor of postoperative explantation rates [OR 24.28, 95% CI 2.81-209.77, p=0.004). Salvage implantation (OR 0.114, 95% CI 0.02-0.67, p=0.016) and non-prostatectomy related incontinence (OR 0.104, 95% CI 0.02-0.74, p=0.023) were independent predictors for worse continence outcomes. Low visual analogue scale scores (OR 9.999, 95% CI 1,42-70.25, p=0.021) and ICIQ-SF scores, respectively (OR 0.674, 95% CI 0.51-0.88, p=0.004) were independent predictors for increased HRQOL outcomes. Perioperative complications did not significantly impact on continence and HRQOL outcomes.

**Conclusion:**

Findings show postoperative infections adversely affect device survival after AUS implantation. However, if explantation can be avoided, the comparative long-term functional results and HRQOL outcomes are similar between patients with or without perioperative complications.

## INTRODUCTION

Current guidelines recommend surgical management of patients with persistent stress-urinary incontinence (SUI) ( 1 - 3 ). Reflective of the high success rates current treatment algorithms recommend the artificial urinary sphincter (AUS) as the gold standard treatment option for persistent moderate-to-severe SUI ( 1 , 3 , 4 ). Even though there are alternative devices available, the AMS 800® (Boston Scientific, USA) is the most frequently used AUS, and low-grade evidence suggests that outcomes may be superior compared to less frequently used devices ( 5 ). Our working group has recently demonstrated that intraoperative complications, postoperative bleeding and urinary tract infection as well as wound healing concerns are independent risk factors for short-term device explantation ( 6 ). However, the study did not evaluate the impact of perioperative complications on long-term functional outcomes. In addition, the inclusion of data from low-volume centers may limit generalized applicability of results ( 6 ).

Perioperative morbidity after AUS implantation is significant as demonstrated by a recent meta-analysis ( 7 ). Despite these findings, the impact of perioperative complications on long-term outcomes after AUS implantation is not fully understood. In this current study, we aim to evaluate the ramifications of perioperative complications on long-term functional and health-related quality of life (HRQOL) outcomes as well as the impact on device survival.

## MATERIALS AND METHODS

### Patient cohort, inclusion and exclusion criteria

The “Debates on Male Incontinence (DOMINO)” database is an international multi-institutional database that includes clinical data from 1047 male patients who have undergone implantation of a continence device due to SUI between 2010 and 2012 in one of 18 regional incontinence surgery referral centers. The inclusion criteria for the current study encompassed the following parameters: Non-neurogenic, moderate-to-severe SUI (≥3 pads) and primary implantation of a single-cuff AUS between 2010 and 2012 in a high-volume center (>100 previous implantations). In total, 105 patients from five different centers were eligible to participate in the current study. The surgical approach followed recommendations by national working groups on male urinary incontinence ( 8 ). The perioperative treatment course including perioperative antibiotic prophylaxis and time to trial without catheter (TWOC) varied slightly between the respective centers.

### Study design, data assessment, definitions

Independent urologists, (not involved with the referral centers), performed the entire data assessment. After approval by a local ethics committee (University of Frankfurt, #442/13), questionnaires were sent per mail and information about the functional outcome was accrued. Medical records were interrogated/reviewed for perioperative complications (postoperative bleeding, wound healing disorders, acute urinary retention, infection, and de-novo urgency) and the perioperative course of action including time to TWOC, antibiotic prophylaxis and therapeutic management. The validated Clavien-Dindo scale was implemented to grade complications ( 9 ). Reporting of surgical complications followed the Martin criteria and is therefore consistent with current urologic guidelines ( 10 , 11 ).

Notably, defined infections were not limited to devices only and included any clinical presentation for fever, local tenderness, erythema and/or abscess. De-novo urgency and acute urinary retention were only considered if requiring interventional management (e.g. catheterization). Scrotal hematoma represented postoperative bleeding. Outpatient data was appraised to gain detailed information about etiology and explantation rates respectively.

The following validated tools were employed to assess functional outcomes: International Consultation on Incontinence Questionnaire in its short form (ICIQ-SF) ( 12 ) and the International Quality of Life (IQOL) score ( 13 ). Continence was defined as the usage of up to a single daily safety pad (dry).

### Statistical analysis

In this study, we assessed device survival, continence outcomes and quality of life. The Chi2 test was applied for categorical data analysis whereas Spearman’s rank correlation and Kruskal-Wallis test evaluated continuous data. A Kaplan-Meier curve was implemented together with log-rank tests to analyze device survival. Multivariate analysis required application of binary logistic regression models. All statistical analyses were performed using SPSS V23.0 (IBM, USA). A p value <0.05 was considered to be statistically significant.

## RESULTS

### Patient characteristics, complications, perioperative treatment courses

Median follow-up was 38 (min 25 - max 58) months. Three out of four deaths during the follow-up period were from non-prostate related causes, a single patient passed away from progressive prostate cancer. There was no recorded procedure-related mortality. Functional outcome data was available for 75.0% of the remaining patients.

Mean duration of perioperative antibiotic treatment was 7.8±4.1 days. 37.5% of the patients received a single-shot antibiotic prophylaxis. Detailed patient characteristics as well as perioperative complications are summarized in Table-1 .

**Table 1:** Patient characteristics of 105 patients that met the inclusion criteria and were included in the current study.

No. of patients	105
**Preoperative patient characteristics**	
Age [yrs; mean±SD]	70.1±7.0
Post-prostatectomy SUI	83 (79.0)
BMI [kg/m2; mean±SD]	27.5±3.8
Pelvic external beam radiation [n (%)]	30 (28.6)
Duration of SUI [yrs; mean±SD]	5.2±4.8
Preoperative daily pad use [mean±SD]	7.0±2.8
**Salvage implantation [n (%)]**	32 (30.5)
Surgical procedure	
Perineal AUS [n (%)]	50 (47.6)
Penoscrotal AUS [n (%)]	55 (52.4)
Operation time [min; mean±SD]	76.3±30.9
Intraoperative complication [n (%)]	6 (5.7)
Catheter indwelling time [d; mean±SD]	2.9±1.0
Hospitalization period [d; mean±SD]	6.3±2.7
**Perioperative** **complications**	
Bleeding [n (%)]	5 (4.8)
Impaired wound healing [n (%)]	5 (4.8)
UTI [n (%)]	8 (7.6)
Urinary retention [n (%)]	10 (9.5)
Pain [VAS >0; n (%)]	9 (8.6)
De-novo urge [n (%)]	4 (3.8)
**Perioperative complications [Clavien scale]**	
Clavien I [n (%)]	18 (17.1)
Clavien II [n (%)]	7 (6.7)
Clavien IIIa [n (%)]	5 (4.8)
Clavien IIIb [n (%)]	35 (33.3)
Clavien IV [n (%)]	0 (0.0)
Clavien V [n (%)]	0 (0.0)

**AUS** = artificial urinary sphincter, **BMI** = body-mass index, **SD** = standard deviation, **SUI** = stress urinary incontinence, **UTI** = urinary tract infection, **VAS** = visual analogue scale

### Explantation rates

Within the follow-up period, 29 devices have been explanted, leading to an explantation rate of 26.7%. The causes for device explantation included urethral erosion (n=12), device infection (n=8), urethral atrophy (n=3), fistula (n=2), device dislocation (n=1), and continence failure (n=1).

Univariate analysis ( Table-2 ) demonstrated postoperative UTI (88.9 vs. 22.6%, p <0.001) as well as any other postoperative complications (60.4 vs. 0.0%, p <0.001) significantly increased explantation rates. In patients with previous pelvic radiation there were no increased explantation rates (30.0 vs. 26.7%, p=0.810).

**Table 2:** Univariate analysis of the effect of selected perioperative complications on postoperative explantation rates, continence rates, and health-related quality of life based on the validated I-QOL score after a median follow-up of 38 months. The I-QOL cut-off score of 93 is based on the median score of the entire cohort.

Complication	Explantation (%)	p value	Continence [%]	p value	IQOL≥93 [%]	p value
Radiotherapy [yes/no]	30.0 / 26.7	0.810	37.3 / 55.7	0.168	60.0 / 77.3	0.609
Intraoperative complication [yes/no]	10.3 / 3.9	0.343	33.3 / 50.0	0.516	66.7 / 47.6	0.608
Bleeding [yes/no]	60.0 / 26.0	0.128	50.0 / 46.8	0.889	0.0 / 50.0	1.000
Wound healing disorder [yes/no]	60.0 / 26.0	0.128	50.0 / 47.8	1.000	0.0 / 50.0	1.000
Urinary retention [yes/no]	50.0 / 25.3	0.135	50.0 / 47.8	1.000	0.0 / 50.0	1.000
Pain [VAS 0 vs. any other]	55.6 / 25.0	0.094	56.7 / 35.3	0.228	65.5 / 18.8	**0.004**
Urinary tract infection [yes/no]	88.9 / 22.6	**<0.001**	33.3 / 50.0	0.667	66.7 / 44.7	0.403
De-novo urge [yes/no]	25.0 / 26.6	1.00	0.0 / 57.8	0.444	0.0 / 45.5	1.000

**VAS** =visual analogue scale

In age-adjusted multivariate analysis UTI was confirmed as an independent predictor of postoperative device explantation [odds ratio (OR) 24.28, 95% confidence interval (CI) 2.81 - 209.77, p=0.004).

### Continence outcomes

We found a mean pad usage of 1.2±1.1 per day, representing a continence rate of 48.4%. 93.8% would recommend the AUS device to a friend and would undergo AUS implantation again. Mean ICIQ-SF score was 7.7±5.0.

The impact of perioperative complications on continence outcomes using univariate analysis is summarized in Table-2 . In summary, we did not observe significantly altered continence rates despite perioperative complications. We found significantly decreased continence rates for patients with non-PPI (60.9 vs. 39.1%, p=0.017) as well as a statistical trend towards decreased continence rates in patients undergoing salvage AUS implantation (59.4 vs. 26.7%, p=0.059).

In multivariate analysis, adjusted for patient’s age, independent predictors for worse continence outcomes were salvage implantation (OR 0.114, 95% CI 0.02 - 0.67, p=0.016) and non-PPI (OR 0.104, 95% CI 0.02 - 0.74, p=0.023).

### HRQOL outcomes

Mean postoperative IQOL score was 84.8±22.5 (median 93). For further analysis of HRQOL outcomes, patients were divided into two groups depending on the respective IQOL score (<93 vs. > 93). In univariate analysis ( Table-2 ), postoperative HRQOL was significantly impacted by postoperative pain based on VAS (“yes” vs. “no”; 65.5 vs. 18.8%, p=0.004). Continent patients were found to have significantly better HRQOL (68.2 vs. 30.3%, p=0.017), ICIQ-SF scores (p <0.001) as well as lower postoperative daily pad usage (p=0.003).

In multivariate analysis adjusted for patient’s age, a VAS pain score of 0 (OR 9.999, 95% CI 1.42 - 70.25, p=0.021) and lower ICIQ-SF scores (OR 0.674, 95% CI 0.51 - 0.88, p=0.004) were confirmed as independent predictors for improved HRQOL outcomes.

## DISCUSSION

The current study investigates the impact of perioperative complications on long-term outcomes after AUS implantation. Our working group has described various complications following AMS 800 and adjustable male sling implantation for moderate-to-severe SUI ( 14 ). This study however further refined the inclusion criteria, limiting accrual to male patients with primary AUS implantation for moderate-to-severe non-neurogenic SUI in high-volume centers between 2010 and 2012. Moreover, this provides a homogenous patient cohort comparative to previous studies. Our comprehensive analysis of investigating continence outcomes after AUS implantation, device explantation rates and HRQOL allows our study to provide a more global view on favorable outcomes and overcome major shortcomings of previous studies ( 6 , 15 ).

In the current study, we assess the impact perioperative complications have on long-term device explantation rates. Hereby, we confirmed previous evaluations regarding the effect of perioperative complications on 90-days explantation rates ( 6 ). In line with previous reports, we observed the most common cause to be postoperative infections. In addition, we found statistical trends towards higher explantation rates after postoperative bleeding, wound healing concerns or urinary retention. Our results are in line with findings of Linder et al., describing adverse short-term device survival after urinary retention. Furthermore cardiovascular disease, body-mass index, history of pelvic external beam radiation and previous invasive incontinence measures did not negatively impact short-term device survival ( 16 ). However, other studies describe a worse outcome or increased complication rates for irradiated patients ( 17 , 18 ). In spite of the major impact perioperative infections has on our contemporary patient cohort, we did not find a significant benefit of the perioperative antibiotic treatment regime (duration of treatment, single-shot prophylaxis) on explantation rates. Despite increasing appreciation for risk factors affecting device infection and consecutive urethral erosion after AUS implantation, evidence regarding optimal perioperative antimicrobial management remains limited ( 19 , 20 ). In a recent review article, Hofer and Gonzalez concluded that strict perioperative antibiotic prophylaxis and sterile surgical technique seem to be crucial for acceptable surgical outcomes ( 21 ). However, the authors did not discuss the optimal duration of antimicrobial prophylaxis treatment. In addition, evidence suggests that antibiotic coating of the AUS does not decrease postoperative device infection rates ( 22 ). Due to the lack of evidence, antimicrobial prophylaxis regimens still vary significantly between institutions.

Naturally, a favorable outcome after AUS implantation implies adequate continence outcomes as well as adequate long-term HRQOL. In this contemporary patient cohort, we observe continence rates (defined as the need for up to one dry safety pad) of 48.4%. These results are in accordance with the 4% to 86% described in a meta-analysis by van der Aa et al. ( 7 ). In assessing predictive factors for functional outcomes, we found significantly worse continence rates for patients undergoing salvage surgery as well as for non-PPI patients. This is partly in contrast with existing literature. Interestingly, a retrospective single-center analysis of 64 patients demonstrated previous invasive incontinence treatments had no significant impact on continence rates following AUS implantation ( 20 ). However, considering previous invasive continence therapies may affect the regenerative potential of tissue within the surgical field, contributing to secondary tissue scarring, the subsequent AUS implantation may be more complex. In addition, patients suffering from non-radical prostatectomy related SUI (e.g.TUR-P or HIFU), may be prone to more severe extrinsic urinary sphincter damage, which in turn may manifest in worse continence results after AUS implantation. At present, possible mechanisms are not fully understood and warrant further investigation in larger patient cohorts.

To our knowledge, this is the first study investigating the impact of perioperative complications during AUS implantation on long-term continence and HRQOL outcomes. Our findings have several clinical implications. Firstly, we confirm the adverse effect of perioperative complications on device survival after AUS implantation. However, long-term continence and HRQOL outcomes seem to be comparatively similar between patients with or without perioperative complications if explantation can be avoided. Therefore, secondly, appropriate patient counseling is of imminent importance.

This study is not devoid of limitations. First and foremost are the limitations that are inherent to retrospective analyses in general. Even though the follow-up was assessed using standardized questionnaires, preoperative diagnostics were based on institutional pathways and not standardized. Furthermore, due to the multi-institutional design of this study, individual learning curves may impact on favorable outcomes ( 23 ). Lastly, the limited sample size of the current study, warrants future studies, with larger patient cohorts, to validate our results.

## CONCLUSIONS

This study investigated data from high-volume continence referral centers, focusing on primary implantation of single-cuff artificial urinary sphincters by adequately experienced surgeons. We observed significantly increased explantation rates for patients with postoperative urinary tract infections. However, despite some perioperative complications the avoidance of explantation did not significantly affect functional outcome or postoperative HRQOL. Due to the small number of postoperative complications, larger studies with higher event rates are needed to confirm these findings.

## ABBREVIATIONS

AUS: artificial urinary sphincter
HRQOL: health-related quality of life
ICIQ-SF: International Consultation on Incontinence Questionnaire short form
IQOL: International Quality of Life score
PPI: Post-prostatectomy incontinence
SUI: stress urinary incontinence
TWOC: time to trial without catheter
UTI: urinary tract infection
